# The electrocardiogram on the wrist: a frightening experience to the untrained consumer: a case report

**DOI:** 10.1186/s13256-023-03806-3

**Published:** 2023-03-05

**Authors:** Michael Zenzes, Philip Seba, Bettina Portocarrero Vivero-Fäh

**Affiliations:** 1 Department Medizin, Kantonsspital Winterthur, 8400 Winterthur, Switzerland; 2 Department for Restorative, Preventive and Pediatric Dentistry, Charité-Universitätsmedizin Berlin, 14197 Berlin, Germany; 3 Notfallzentrum für Erwachsene, Kantonsspital Winterthur, 8400 Winterthur, Switzerland

**Keywords:** Smartwatch, ECG, Side effects, Anxiety, Case report

## Abstract

**Background:**

Smartwatches offering electrocardiogram recordings advertise the benefits of supporting an active and healthy lifestyle. More often, medical professionals are faced with privately acquired electrocardiogram data of undetermined quality recorded by smartwatches. This is boasted by results and suggestions for medical benefits, based on industry-sponsored trials and potentially biased case reports. Yet potential risks and adverse effects have been widely overlooked.

**Case presentation:**

This case report describes an emergency consultation of a 27-year-old Swiss–German man lacking known previous medical conditions who developed an episode of anxiety and panic due to pain in the left chest prompted by over-interpretation of unremarkable electrocardiogram readings of his smartwatch. Fearing acute coronary syndrome, he presented at the emergency department. His smartwatch electrocardiograms, as well as a 12-lead electrocardiogram, appeared normal. After extensive calming and reassuring, as well as symptomatic therapy with paracetamol and lorazepam, the patient was discharged with no indications for further treatment.

**Conclusions:**

This case demonstrates the potential risks of anxiety from nonprofessional electrocardiogram recordings by smartwatches. Medico–legal and practical aspects of electrocardiogram recordings by smartwatches need to be further considered. The case shows the potential side effects of pseudo-medical recommendations for the untrained consumer, and may add to the discussion on the ethics of how to evaluate smartwatch electrocardiogram data as a medical professional.

## Background

Smartwatches are becoming increasingly popular as technical support for a healthy lifestyle. Many devices include components of self-tracking and self-optimization of daily activities based on measurements of heart rate, oxidation level, activity, and stress indicators. Reports of shortcomings and side effects have, however, been scarce. The most popular smartwatch for example, currently produced by Apple (Cupertino, USA) [1], offered heart rate tracking since its first series, and fourth generation devices (since 2018) include a single-lead electrocardiogram (ECG) function, most comparable to Einthoven I. Thirty-second rhythm strips obtained by the watch are stored and used for further analysis and possible diagnoses. The ECG app provides one of the following analysis results: sinus rhythm, atrial fibrillation, atrial fibrillation with high heart rate, or poor recording.

Medical professionals are increasingly confronted with ECGs of unknown reliability from non-medically certified smartwatches. The possible implications for outpatient monitoring or diagnostics are subject to current discussions and research. As described in this case report, unsupervised ECGs acquisitions may cause side effects such as anxiety, while entailing a false feeling of security. The untrained consumer cannot judge the diagnostic capacity and shortcomings of a single-lead ECG despite the support of intelligent analysis software. Procedures to follow in case of a warning or abnormal analysis are unclear (emergency consultation, urgent consultation within office hours, or regular follow-up visit) unless arranged in advance with a medical professional.

Current literature discusses the role of smartwatches in the early detection of atrial fibrillation or flutter, the most common forms of arrhythmia that have increasing prevalence with older age [2–5]. Long-term, continuous recordings of ECGs are the typical domain of the classic Holter ECG or implantable loop devices. The increasing availability and distribution of ECG-capable wearables, data storage via a cloud, and location-independent use may offer innovative potential and benefits for detecting asymptomatic arrhythmias. Smartwatches may also complement current telemedical services with interesting potential, to the extent that the European Heart Rhythm Association (EHRA) of the European Society of Cardiology (ESC), issued practical guidelines on digital devices and the detection of arrhythmias [6].

This report concerns a patient that admitted himself as a result of self-interactions with his smartwatch. Since our patient used an Apple watch, we take this manufacturer as an example for the article. The detection of atrial fibrillation using photoplethysmography (PPG), as employed by the Apple watch for heart rate measurement, has shown high sensitivity of 93.7% and specificity of 98.2% [5]. Yet 21.8% of the data was not interpretable due to poor signal quality [5]. This matches reports that the sensor is prone to errors with incomplete skin contact, dark skin, or motion [7]. In a cohort of more than 400,000 participants using an Apple watch, 0.52% of the entire study population versus 3.2% of participants above the age of 65 years received an “irregular rhythm notification” by the app after four detected episodes of irregular rhythm. 404 notified participants confirmed newly diagnosed atrial fibrillation, while 3070 newly diagnosed participants received no notification by the app. 76% of notified participants consulted a medical professional, and 15 out of a total of 1038 adverse events (~ 1.5%) were anxiety related [4].

A retrospective analysis reported an 11.4% incidence of newly diagnosed cardiovascular conditions after consultations due to irregular rhythm notifications by the Apple watch [8]. The probability of false-positive warnings was especially high in a young population without cardiovascular risk factors [4, 8]. False-positive notifications resulted in unnecessary consultations [8].

The diagnosis of acute coronary syndrome (ACS) using an Apple watch [9], described by one case report, is an “off-label” application, which is neither intended by the manufacturer, nor US Food and Drug Administration (FDA) approved. A sequential three-lead ECG can be obtained by placing an Apple watch on different extremities [10], which may enable early, preclinical diagnosis of ST-elevation myocardial infarctions.

There is uncertainty among medical professionals how reliable the PPG and ECG recordings of different manufacturers and wearables actually are [7, 11]. To ensure reliability and safety, medical devices undergo a complex process of authorization and validation. The requirements for testing and authorization in the European Union are defined by the medical device regulation (MDR) EU 2017/754 and are necessary for CE certification and distribution on the common market [12]. A CE certification for risk category I, obtained by the manufacturer, was approved for the Apple watch feature “irregular heart rhythm notification” and the “ECG app” in March 2019 [13]. The apps are considered medical software but the watch is not required to be certified as a medical device, since the ECG app can be installed and removed.

Despite the rapidly evolving field and possible benefits that smartwatches offer to the medical area, the technology is still new, and thus merits further considerations to find valuable and safe applications.

## Case report

A 27-year-old, Swiss–German man presented himself at the emergency service during night hours. After a sports competition during the day, he noticed a movement-dependent, stabbing pain in his left chest. Fearing an ACS, he recorded multiple, repeated, single-lead ECGs with his Apple watch. The patient purchased the smartwatch to monitor his “cardiac health” and check for arrhythmias after media coverage of a Danish soccer player experiencing cardiac arrest during a match [14]. Prior to the event, the patient had never experienced palpitations or arrhythmias.

The ECG app attested a “normal sinus rhythm” for each of his multiple ECG recordings. The Apple watch regularly indicates, during ECG recording, that it cannot detect heart attacks. Yet the stabbing pain persisted. Despite the automated warnings that the smartwatch cannot diagnose ACS, the patient developed anxiety and began online research for ECG changes in ACS. He came across a description of hyper-acute T-waves as an indication of ACS and interpreted the pronounced T-wave on his single-lead ECG as such. He felt significantly worse, uneasy, and developed difficulty breathing. Upon consultation at the emergency department, the patient was extremely worried and described worsening symptoms of anxiety and panic, including tachycardia, palpitations, and perspiration. He had not taken any pain medication for the motion-dependent pain.

The young man was fit and healthy, a university student living with his girlfriend, reporting recreational use of tobacco. His history was unremarkable for preexisting medical or mental diagnosis, regular medication, further cardiovascular risk factors, or a family history of cardiovascular or mental conditions. There was no relevant surgical history. He described that he sometimes “gets stuck in his head” when he worried about something, but he was always able to calm and control himself. He never required professional psychotherapeutic support. The man denied the use of recreational or performance-enhancing drugs. The physical examination was unremarkable.

A 12-lead ECG in the emergency department showed a sinus rhythm of 88 bpm with normal conduction times and no indication of ischemia. The morphology and amplitude of the T-waves were normal in all 12 leads.

Due to the low pre-test probability, the unremarkable history, and the clinical presentation, no further diagnostic tests were justified. There was no other evident cause for the anxiety besides the over-interpretation of the single-lead ECG in combination with his post-exertional musculoskeletal pain. After administration of 500 mg of paracetamol (acetaminophen) and 1 mg of lorazepam, the movement-dependent, stabbing pain subsided, and the patient felt substantially better.

After discussing the limited value of a single-lead ECG for the diagnosis of myocardial infarction and the presentation of the different T-wave morphologies in the 12-lead ECG, the patient was discharged from the emergency department in symptom-free condition. Further recommendations were given in case of recurrent chest pain or anxiety and panic. Consultation with the family doctor was suggested to evaluate (a) the need for cardiological evaluation or (b) specific psychotherapy. We also encouraged him to stop smoking.

In a follow-up telephone interview for this report, the patient reported an unremarkable cardiological evaluation in private practice, as well as a consultation with an osteopath to improve the mobility of his torso and learn breathing techniques. He stopped smoking in the meantime. He feels fit and healthy and continues his competitive sportive activities. The patient did not use his Apple watch again.

## Discussion and conclusion

ECG, a diagnostic tool normally available under the supervision of a medical professional, has become broadly accessible to the public. Smartwatches, advertised as an adjunct for a healthy lifestyle, are not subject to an external conformity assessment as medical products. Considering a realistic wearing duration of more than 60 minutes per day, a smartwatch may be classified in risk category IIa—the same as, for example, a hearing aid. According to the MDR, this would result in the need for external conformity assessment.

Despite their theoretical medical benefits, smartwatches lack sufficient scientific evidence of their clinical benefits for the general population. The data acquired by PPG may not be sufficient to detect all cases of arrhythmia. In certain situations, a smartwatch ECG appears to be more accurate in this regard [6].

Notwithstanding the practical guidelines, it remains unclear how to implement the data for diagnostics or treatment surveillance. Randomized controlled trials evaluating the clinical benefit are urgently needed. The use and reliability of smartwatches must be judged “in hindsight” by the treating medical personnel based on the individual case and patient. There is a risk of unnecessary invasive and potentially harmful diagnostic procedures to “rule out” diagnoses raised by the smartwatch. Potential adverse psychological effects, such as anxiety or panic in susceptible individuals, have been largely under-reported.

This case report has limitations. The treatment of post-exertional musculoskeletal pain was straightforward. There was no justified call for extensive, invasive diagnostics or follow-up visits. We can only assume that the phenomenon could have occurred similarly with other smartwatch manufacturers. The over-interpretation of a single-lead ECG in combination with musculoskeletal pain led to an episode of anxiety, a feeling of illness, and an unnecessary emergency consultation during the night. We believe that clinicians and emergency department teams should be aware of such cases. With an unknown risk-to-benefit ratio there appears to be a need for further research to avoid side effects of potentially beneficial smartwatch accessories. Awareness can likely lead to the introduction of measures such as adding an algorithm that explores the benefits of family doctor intervention or starting a biofeedback application to relax and distract the user if a certain threshold of ECG measurements per hour is exceeded.

As shown by the present case report, untrained users may over-interpret the results of the ECG function beyond the intended application, despite automated warnings. This will inevitably lead to wrong conclusions, may trigger anxiety, and causes unnecessary consultations. It remains unclear how meaningful training or information of the individual consumer—beyond automated warnings or liability exclusions—may look in the future. Ideally, the usage of a smartwatch should be discussed with a medical professional and implemented in a management plan.

A medical specialist in the office or clinic should handle the diagnosis and therapy of arrhythmias and ACS, for which an approved 12-lead ECG confirmation is mandatory. To use the resources of the public healthcare sector economically, to avoid unnecessary consultations and potentially harmful invasive diagnostic tests, there is room for a management plan and diagnostic algorithm to distinguish “false positive” users from possible patients requiring further investigation and care.

Medical professionals must learn how to deal critically yet adequately with privately collected, nonregulated, unvalidated ECG data obtained by smartwatches to provide comprehensive care and consultation. It appears that this rapidly evolving field can no longer be dismissed by the medical teams that are going to be confronted by future technical developments, their potential benefits, and risks.

## Data Availability

Not applicable.

## Abbreviations

ECG: Electrocardiogram
PPG: Photoplethysmography
ACS: Acute coronary syndrome
FDA: United States Food and Drug Administration
MDR: Medical device regulation
CE: Conformité Européenne
Bpm: Beats per minute
